# Cost-effectiveness of hepatitis C virus screening, and subsequent monitoring or treatment among pregnant women in the Netherlands

**DOI:** 10.1007/s10198-020-01236-2

**Published:** 2020-10-16

**Authors:** Job F. H. Eijsink, Mohamed N. M. T. Al Khayat, Cornelis Boersma, Peter G. J. ter Horst, Jan C. Wilschut, Maarten J. Postma

**Affiliations:** 1 Unit of PharmacoTherapy, Epidemiology and Economics, Groningen Research Institute Pharmacy, University of Groningen, Groningen, The Netherlands; 2 Department of Economics, Econometrics and Finance, Faculty of Economics and Business, University of Groningen, Groningen, The Netherlands; 3 Department of Health Sciences, University Medical Center Groningen, University of Groningen, Groningen, The Netherlands; 4 Department of Medical Microbiology, University Medical Center Groningen, University of Groningen, Groningen, The Netherlands; 5 Department of Clinical Pharmacy, Isala, Zwolle, The Netherlands

**Keywords:** Hepatitis C virus, Pregnant women, HCV screening, Direct-acting antivirals, C00, C02, C3, C30, C31, I00, I1, I10, I19, H00, H61

## Abstract

**Background:**

The prevalence of diagnosed chronic hepatitis C virus (HCV) infection among pregnant women in the Netherlands is 0.26%, yet many cases remain undiagnosed. HCV screening and treatment of pregnant HCV carriers could reduce the burden of disease and limit vertical transmission from mother to child. We assessed the impact of HCV screening and subsequent treatment with new direct-acting antivirals (DAAs) among pregnant women in the Netherlands.

**Methods:**

An HCV natural history Markov transition state model was developed, to evaluate the public-health and economic impact of HCV screening and treatment. Besides all 179,000 pregnant women in the Netherlands (cohort 1), we modelled 3 further cohorts: all 79,000 first-time pregnant women (cohort 2), 33,000 pregnant migrant women (cohort 3) and 16,000 first-time pregnant migrant women (cohort 4). Each cohort was analyzed in various scenarios: *i no intervention*, i.e., the current practice, *ii screen-and-treat*, i.e., the most extensive approach involving treatment of all individuals found HCV-positive, and *iii screen-and-treat/monitor*, i.e., a strategy involving treatment of symptomatic (F1–F4) patients and follow-up of asymptomatic (F0) HCV carriers with subsequent treatment only at progression.

**Results:**

For all cohorts, comparison between *scenarios *(ii) and (i) resulted in ICERs between €9,306 and €10,173 per QALY gained and 5 year budget impacts varying between €6,283,830 and €19,220,405. For all cohorts, comparison between *scenarios *(iii) and (i) resulted in ICERs between €1,739 and €2,749 per QALY gained and budget impacts varying between €1,468,670 and €5,607,556. For all cohorts, the ICERs (*scenario iii* versus *ii*) involved in delayed treatment of asymptomatic (F0) HCV carriers varied between €56,607 and €56,892, well above the willingness-to-pay (WTP) threshold of €20,000 per QALY gained and even above a threshold of €50,000 per QALY gained.

**Conclusion:**

Universal screening for HCV among all pregnant women in the Netherlands is cost-effective. However, it would be reasonable to consider smaller risk groups in view of the budget impact of the intervention.

**Electronic supplementary material:**

The online version of this article (10.1007/s10198-020-01236-2) contains supplementary material, which is available to authorized users.

## Introduction

Hepatitis C is a serious disease caused by infection with hepatitis C virus (HCV). Worldwide 80–130 million people are chronically infected with HCV [1, 2]. Exposure to the virus results in 80% of cases in a chronic infection [3]. Approximately 20% of chronically infected patients develop serious HCV-related liver disease after onset of the infection [4]. Currently, hepatitis C affects 8% of pregnant women globally [5]. HCV may be transmitted vertically, mostly perinatally, from mother-to-child [6–8]. With the development of new drug therapies which are highly effective and well tolerated, there is a potential for these drugs to be used by pregnant patients with hepatitis C [9]. HCV screening of pregnant women potentially contributes to the goal of the World Health Organization (WHO) to achieve 90% diagnosis of HCV and 80% treatment by 2030 worldwide through scaling-up screening strategies and prevention of HCV transmission [10].

Two major developments have contributed to the demand for HCV screening of specific risk groups. The first and most important development is the improved HCV treatment with direct-acting antivirals (DAAs) [11]. More than 90% of chronically infected HCV patients are cured through DAA treatment compared to only 50% with previous treatments [12–14]. The second development is the increase of hepatocellular cancer (HCC) incidence, HCV infection being the leading cause of HCC in western countries [15]. Screening and DAA treatment of risk groups could prevent reinfection, new infections, HCC and vertical transmission from mother-to-child.

The Health Council in the Netherlands has recommended to investigate the cost-effectiveness of screening of pregnant women for HCV with subsequent DAA treatment [16–18]. Prevalence of diagnosed chronic HCV infection among women in the Dutch population is 0.26% (95% confidence interval (CI): 0.15–0.46%), which is similar to the prevalence in the general population in Europe [17]. First-generation non-western migrants are more likely to be HCV-positive (0.7–2.3%) than western women (0.1–0.4%) [18]. Notably, these immigrants represent 5.9% of the total Dutch female population [19].

In industrialized countries, HCV is the most common cause of chronic liver disease among children and perinatal transmission is the leading cause of infection [20]. The current best estimate of vertical transmission risk is between 4.5 and 7.1% [21]. Treatment with DAAs during pregnancy is not yet recommended, and lactation during treatment is contra-indicated, because of a lack of information on potential toxicity [22]. However, it is conceivable that in the near future DAA treatment of HCV-infected women during pregnancy becomes available, not only to limit disease progression in the patient, but also to prevent vertical transmission of the virus to the child.

The aim of this study is to estimate the public-health and economic impact of HCV screening and treatment among pregnant women from a public-health perspective [23]. In particular, we estimated the health gains, cost-effectiveness and the budget impact of implementing such a programme. The results of our study can be used to reach a rational decision as to whether HCV screening and potential treatment of pregnant women should be implemented in the Netherlands and elsewhere [24].

## Methods

### Overview

A screening model linked to HCV-disease states within a Markov model was used to evaluate the cost-effectiveness (CE) of HCV screening of pregnant women, with initial treatment during pregnancy, compared to current practice (no screening and no intervention) from a health-care payer perspective in the Netherlands. Our CE analysis includes health benefits for pregnant women and their children, and the corresponding budget impact. The costs and effects of HCV screening and various modalities of subsequent treatment versus current practice were calculated for four cohorts of pregnant women and were expressed in terms of incremental cost-effectiveness ratio (ICER), as further elaborated below.

### Model

**Fig. 1:**
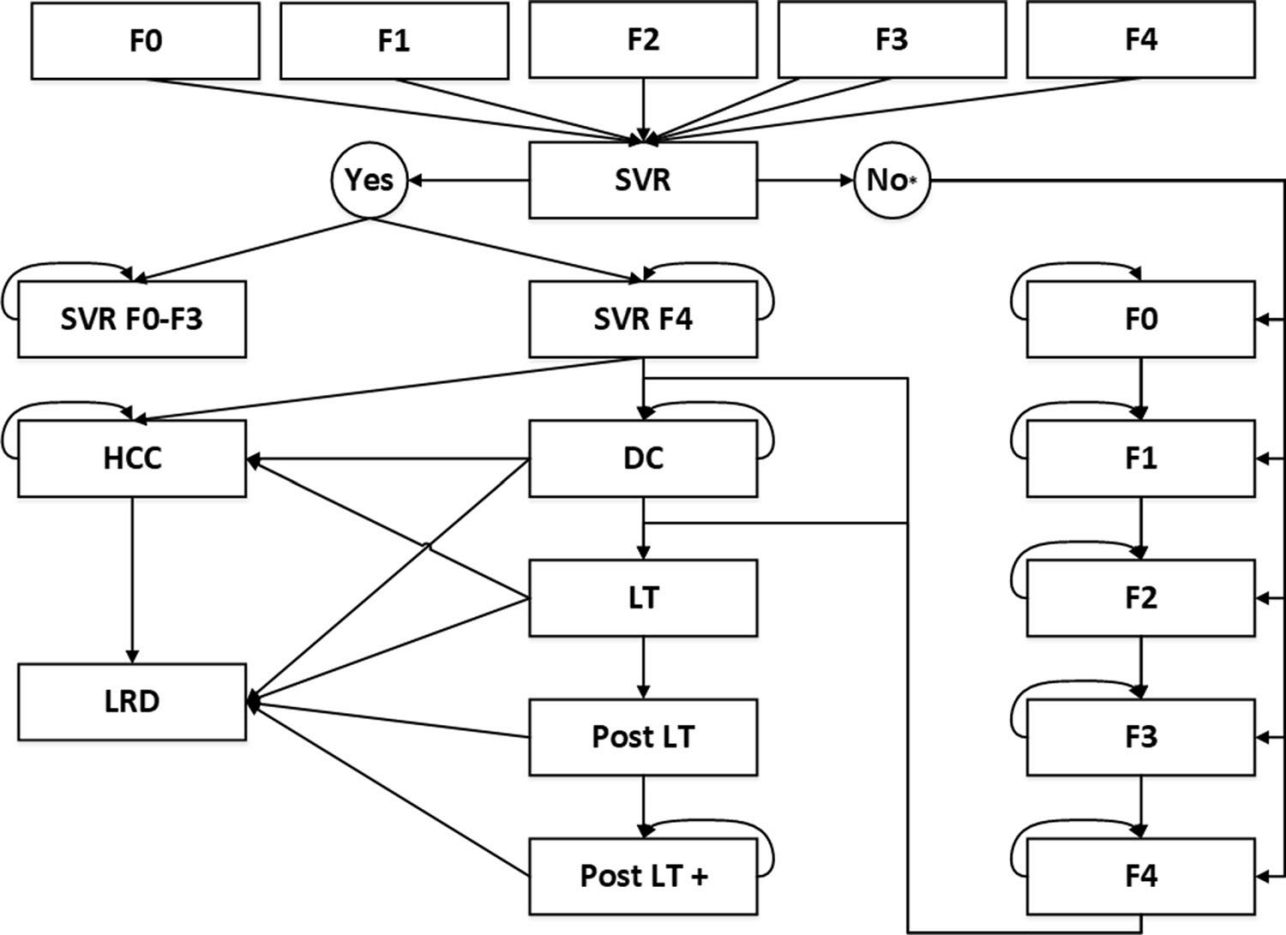
Markov Model for chronic hepatitis C progression of disease. METAVIR score: F0, F1, F2, F3, F4. SVR: sustained virologic response. *HCC* hepatocellular cancer, *DCC* decompensated cirhossis, *LT* liver transplantation. LRD: liver-related death. *In case of treatment failure, patients will be in the same METAVIR state after the treatment

We used a deterministic, HCV natural history, closed-cohort Markov Model, as presented in Fig. 1. The model includes annual cycles and a life-time horizon of 70 years, representing the approximate period from the age at which a woman can become pregnant until her death. HCV carriers were classified in METAVIR scores F0–F4. F0 is a (fully) healthy, but HCV-infected, state. F1-F3 represent mild to severe stages of liver fibrosis. F4 represents liver cirrhosis. In the model, patients with METAVIR score F4 may develop hepatocellular cancer (HCC), decompensated cirrhosis (DCC) and, subsequently, patients with DCC can progress to liver transplantation (LT). LT-patients move to the follow-up state (post-LT). Post-LT patients are described as patients during the first 12 months after their liver transplantation. After 1 year, they move to the follow-up state post-LT + until their death. Without screening, HCV-infected patients generally develop symptoms in a late stage of infection [18, 25]. Implementation of screening will result in detection of increased numbers of asymptomatic patients [26] and, later on, fewer patients with fibrosis or cirrhosis relative to the current situation without screening. In this study, we assumed that testing a cohort comprising of all pregnant women is a 'one-time' screening for each women (independent of the number of pregnancies), rather than having repeat testing in their potential subsequent pregnancies.

In the model, we used a conservative sustained virologic response (SVR) of 95% for patients with METAVIR scores of F0, F1, F2 and F3, and 90% for F4 patients [11]. We only included treatment regimens for 12 weeks, independent on the METAVIR scores and in accordance with the Dutch HCV-guidelines [27, 28]. It was assumed that if patients were not cured, they proceed to the next lower health state. Vertical transmissions are included in the model as potentially prevented HCV infections, after screening of the mothers and subsequent DAA treatment. The probabilities to move from one health state to another are given in Table S1 of the Appendix.

### Hepatitis C virus testing

The first HCV screening step represents a serologic antibody test to determine the presence of a current or past HCV infection. The second test is a reverse-transcription polymerase-chain reaction (RT-PCR) viral RNA test to confirm the serologic test, and to determine whether the HCV infection had been cleared spontaneously. The RT-PCR test has a sensitivity between 61.0% and 81.8% and a specificity between 97.5 and 99.7% [29]. The third test concerned a fibroscan examination, which is a quantitative analysis technique to support the diagnostics of liver fibrosis in patients and to determine the METAVIR score (F0-F4). Individuals are screened first for anti-HCV antibodies and, if found positive, are subsequently screened for HCV RNA. Outpatient visit consultation costs were included for each test. For individuals who are RNA-positive, we incorporated fibroscan costs for disease staging.

### HCV treatment

The annual costs for DAA treatment were assumed at their list price levels in the Netherlands [30]. Weighted average treatment costs for DAA were estimated at €34,000 based on actual use of DAA medication (Sofosbuvir, Ledipasvir/Sofosbuvir, Grazoprevir/Elbasvir, Velpatasvir/Sofosbuvir, Daclatasvir, Ombitasvir/Paritaprevir/Ritonavir) for a 12-week treatment period in 2018 [31]. We did not consider other treatments for HCV infection, such as protease inhibitors, ribavirin or PEG-interferons. For the budget-impact analysis, we included total medical costs in the first 5 years, costs of HCV treatment, screening costs and follow-up costs with possible HCV-related diseases.

### Cohorts of pregnant women in the Netherlands included in the model

The pregnant women included for HCV screening in this study are between 20 and 45 years of age, with an average age of 29 [19, 32]. We excluded women with recurrent HCV infection, women with HIV infection and injecting drug users [33]. In this study, we considered four different cohorts of pregnant women. The characteristics of the four cohorts were obtained from Statistics Netherlands (CBS); we took the average size of the years 2000 to 2017. Details of the cohorts, including size, HCV prevalence and vertical transmission estimates [21], are as follows:

(i) All Dutch pregnant women, with an HCV prevalence of 0.26% and cohort size of 179,000, with 465 HCV cases and 27 (range 22–32) vertical transmissions.
(ii) All Dutch pregnant women during first-time pregnancy, with an HCV prevalence of 0.37% and cohort size of 79,200, with 292 HCV cases and 18 (range 14–21) vertical transmissions.
(iii) First-generation non-western pregnant migrants, with an HCV prevalence of 0.70% and a cohort size of 33,000, with 231 HCV cases and 14 (range 11–16) vertical transmissions.
(iv) First-generation non-western pregnant migrants during first-time pregnancy with an HCV prevalence of 1.0% and a cohort size of 16,000, with 160 HCV cases and 10 (range: 8–11) vertical transmissions.

### Utilities of HCV health states

Quality of life depends on the state of health and the age of the pregnant woman. In the model, all HCV health states were assigned a particular utility, ranging from 0 to 1. Utility 0 reflects death and utility 1 reflects full health without any complaints. The utility of HCV-positive, but asymptomatic, patients (F0) was reduced with 0.02 [34, 35], because of reasons of anxiety and worries and among these individuals. Utilities after successful treatment were assumed to increase by 0.05 [36]. The utilities are presented in S1 of the Appendix. Quality-adjusted life years (QALYs) were calculated as the product of remaining life years of the patient in a particular health state after the intervention (screening and monitoring or treatment) and the quality of life after the intervention [36].

### Scenarios

We investigated three scenarios with different comparisons between the scenarios. *Scenario i, the no-intervention* scenario, reflects the current practice of absence of screening. *Scenario ii*, the *screen-and-treat* scenario, reflects the most extensive approach with DAA treatment of all individuals found HCV-positive after screening. Finally, *scenario iii*, the *screen-and-treat/monitor* scenario, reflects the approach in which, after screening, the F0 patients are not treated but actively monitored (and, if indicated, treated later on). We specifically considered this third scenario to avoid delayed overtreatment. Indeed, 20% of asymptomatic HCV-infected individuals spontaneously clear the virus and, in addition, approximately 80% of chronically infected patients will never develop HCV-related liver disease [37]. Obviously, one does not know a priori which patients will develop chronic infection and symptoms of disease. Therefore, we chose to periodically monitor these patients. We assumed that just monitoring asymptomatic HCV carriers instead of treatment would contribute to lower treatment costs and result in higher patient value.

Three comparisons between the different scenarios were performed:

- *Scenario ii* versus *scenario i*, reflecting screening and treatment of all HCV-positive patients versus the current practice of no intervention.
- *Scenario iii* versus *scenario i*, reflecting treatment of symptomatic (F1–F4) patients and monitoring of asymptomatic (F0) HCV carriers versus the current practice.
- *Scenario iii* versus *scenario ii,* focusing specifically on the additional costs and health gains due to immediate treatment of all F0 HCV carriers versus just monitoring these asymptomatic individuals until some of them progress to disease.

As avoidance of mother-to-child transmission of HCV is one of the most important reasons for HCV screening and treatment of pregnant women, vertical transmissions are explicitly taken into account in the model. Specifically, we included the effects of vertical transmission on the healthcare costs, treatment costs and QALYs for the (unborn) children.

### Incremental cost-effectiveness ratio and budget impact analyses

We express the cost-effectiveness of the different scenarios described above in terms of incremental cost-effectiveness ratio (ICER), using the following formula:

$${\text{ICER}} = \left( {C_{1} - C_{0} } \right)/\left( {E_{1} - E_{0} } \right),$$

in which C represents the costs and E the quality-adjusted life years (QALYs); subscript 1 represents the case where the intervention has been applied and subscript 0 represents the case where the intervention has not been applied. Therefore, the ICER represents the costs per quality-adjusted life year (QALY) gained. In the Netherlands, ICERs are considered against an informal willingness-to-pay (WTP) threshold of €20,000 per QALY gained [38]. Notably, we also considered a WTP-threshold of €50,000 per QALY gained, reflecting the burden of disease [39].

The budget-impact analysis gives a perspective on total future HCV-related costs. For the budget-impact analysis, we included direct medical costs, costs of HCV treatment and costs of screening, in the first 5 years, 10 years and 15 years of implementation of screening according to the budget impact guidelines [40]. The total costs were discounted with an annual rate of 4%, the QALYs were discounted with 1.5%, according to Dutch guidelines [41]. Price levels in the year 2018 were applied.

### Sensitivity analyses

A one-way sensitivity analysis was performed to estimate the effect of variation in specific parameters on the ICER and to determine which parameter has the most pronounced effect on the ICER. The parameters were varied between minus 10% and plus 10% of the base-case parameter value. The prevalence was varied in the range of the 95% CI of the HCV prevalence of 0.26% (0.15–0.46%).

A probabilistic sensitivity analysis (PSA) was performed to assess the uncertainty around the different input parameters and the effect on the CER. Here, input parameters are considered as random quantities based on the underlying parameter distributions. For every simulation (5000 in total), the parameters were sampled from the parameter space of 95% CI. If the 95% CI was unknown for a specific parameter, we varied the parameter between minus 10% and plus 10%, following a triangle distribution. All variables and ranges are represented in Table S2 of the Appendix.

## Results

### Health benefits due to HCV screening and treatment

We first determined the health benefits involved in implementation of HCV screening and DAA treatment among pregnant women in The Netherlands. In all four cohorts, we found significant reductions in liver disease after 2–3 decades, specifically a reduction of 30% in DCC, of 37% in HCC, of 27% in liver transplantation (LT) and of 34% in liver-related death (LRD). We also found significant reductions in vertical HCV transmissions. Since each cohort consists of a different number of pregnant women with a specific HCV prevalence, the absolute number of avoided vertical transmission varied between the different cohorts. Specifically, in the cohort of all pregnant women, we found 27 avoided cases of vertical transmission, in the cohort of first-time pregnant women 18 avoided cases, in the cohort of pregnant migrants 14 avoided cases, and in the cohort of first-time pregnant migrants 10 avoided cases.

### Cost-effectiveness and budget-impact of HCV screening and treatment

We subsequently determined the cost-effectiveness and budget impact of HCV screening and treatment among the four cohorts of pregnant women following the different scenarios and comparisons. Table 1 presents an overview of the results. For each of the cohorts, the table shows the values of the ICER for comparisons between the two respective intervention scenarios and the scenario; *no intervention* (current practice), Table 1 also presents the ICERs for scenario *screen-and-treat* versus *screen-and-treat/monitor* and in Table 2 the total 5 years, 10 years and 15 years budget impact (BI) of the different interventions. Below, we further elaborate on the results obtained for each of the cohorts.

**Table 1:** The total costs, QALYs, incremental costs, QALYs gained and cost-effectiveness (ICERs) of three different scenarios in four pregnant cohorts in the Netherlands: incremental costs and QALYs and ICERs reflect the comparison with the previous scenario, except for the values provided between parenthesis, which compare the respective last and first scenarios (*screen-and-treat* versus *no intervention*)

Cohort	Scenario	Total cost	QALYs	Incremental cost	QALYs gained	ICER
All pregnant women	No intervention	€1,545,141	1362	n.a	n.a	n.a
	Screen-and- treat/monitor	€6,124,234	3028	€ 4,579,093	1666	€2749
	Screen-and-treat	€21,200,440	3294	€15,076,206 (€19,655,299)	266 (1932)	€56,677 (€10,173)
First-time pregnant women	No intervention	€948,419	834	n.a	n.a	n.a
	Screen-and-treat/monitor	€3,397,186	1857	€2,448,767	1023	€2393
	screen-and-treat	€12,623,974	2021	€9,226,788 (€11,675,555)	164 (1187)	€56,260 (€9834)
All pregnant migrant women	No intervention	€782,804	691	n.a	n.a	n.a
	Screen-and- treat/monitor	€2,349,137	1535	€1,566,333	844	€1857
	screen-and-treat	€10,006,625	1669	€7,657,488 (€9,223,821)	135 (978)	€56,722 (€9431)
First-time pregnant migrant women	No intervention	€531,200	468	n.a	n.a	n.a
	Screen-and- treat/monitor	€1,527,568	1041	€996,368	573	€1739
	screen-and-treat	€6,710,574	1132	€5,183,006 (€6,179,374)	92 (664)	€56,337 (€9306)

*ICER* incremental cost-effectiveness ratio, *na* not applicable, *QALY* quality-adjusted life year; (..) comparison between *screen-and-treat* versus *no intervention*

**Table 2:** Budget impact analysis at 5, 10 and 15 years of HCV screen-and-treat scenario and screen-and-treat/monitor scenario, among four different cohorts of pregnant women in the Netherlands

	BI 5 years	BI 10 years	BI 15 years
All pregnant women			
Screen-and-treat/monitor	€5,607,556	€5,893,455	€6,132,468
Screen-and-treat	€19,220,405	€19,370,568	€19,498,491
First-time pregnant women			
Screen-and- treat/monitor	€2,691,789	€2,920,865	€3,112,373
Screen-and-treat	€11,329,356	€11,495,996	€11,737,955
All pregnant migrant women			
Screen-and-treat/monitor	€1,734,575	€1,781,845	€1,823,324
Screen-and-treat	€9,323,994	€9,451,528	€9,560,174
First-time pregnant migrant women			
Screen-and-treat/monitor	€1,468,670	€1,472,211	€1,558,772
Screen-and-treat	€6,283,830	€6,371,987	€6.547,087

*BI* budget impact

#### All pregnant women

The *screen-and-treat* scenario versus *no intervention*, involving treatment of all HCV carriers (F0-F4), and *current practice*, in the cohort of all pregnant Dutch women yielded 1932 QALYs with incremental costs estimated at €19,655,299, resulting in an ICER of €10,173 per QALY gained. The associated total BI at 5 years of this intervention was estimated at €19,220,405, €19,370,568 over 10 years and €19,498,491 over 15 years (Table 2). The second comparison involved the more restrictive *screen-and-treat/monitor* scenario versus *no intervention*. This comparison resulted in a gain of 1666 QALYs with incremental costs estimated at €4,579,093, resulting in a considerably lower ICER of €2749 per QALY gained. The total BI 5 years of this comparison was also considerably lower than that of scenario screen-and-treat at an estimated €5,607,556, €5,893,455 over 10 years and €6,132,468 over 15 years (Table 2).

#### First-time pregnant women

In the cohort of all first-time pregnant women, comparison of the *screen-and-treat* scenario with the *no-intervention* yielded 1187 QALYs at estimated incremental costs of €11,675,555, resulting in an ICER of €9834 per QALY gained. The total BI of this scenario was estimated at €11,329,356 over 5 years, €11,495,996 over 10 years and €11,737,955 over 15 years (Table 2). The second comparison between *screen-and-treat/monitor* and *no intervention* resulted in 1023 QALYs gained with estimated incremental costs of €2,448,767 and an ICER of €2393 per QALY gained. The estimated total budget impact of this scenario was €2,691,789 over 5 years, €2,920,865 over 10 years and €3,112,373 over 15 years. (Table 2).

#### All pregnant migrant women

Within the smaller cohort of pregnant migrants, comparison of the *screen-and-treat* scenario among all HCV carriers with *no intervention*, resulted in 978 QALYs gained and estimated incremental costs of €9,223,821, resulting in an ICER of €9,431per QALY gained. The total budget impact of this screening scenario was estimated at €9,323,994 over 5 years, €9,451,528 over 10 years and €9,560,174 over 15 years (Table 2). Comparison between the *screen-and-treat/monitor* and the *no intervention* scenarios in this cohort yielded 844 QALYs gained at estimated incremental costs of €1,566,333, resulting in an ICER of €1857 per QALY gained. The total BI of this scenario was estimated at €1,734,575 over 5 years, 1,781,845 over 10 years and 1,823,324 over 15 years (Table 2).

#### First-time pregnant migrant women

Limiting the intervention to the cohort of first-time pregnant migrants further improved cost-effectiveness results, with the most favorable outcomes for the *screen-and-treat/monitor* scenario. Specifically, comparison in this group between the *screen-and-treat* and *no intervention* scenarios yielded 664 QALYs gained at incremental costs of €6,179,374, resulting in an ICER of €9,306 per QALY gained. The total BI over 5 years of this screening scenario was estimated at €6,283,830, €6,371,987 over 10 years and €6.547,087 over 15 years (Table 2). The *screen-and-treat/monitor* scenario yielded 573 QALYs gained at incremental costs of €996,368, resulting in an ICER of €1,739 per QALY gained. The total BI of this screen and monitor/treat scenario was estimated at €1,468,670 over 5 years, €1,472,211 over 10 years and €1,558,772 over 15 years (Table 2).

### Effects of delayed treatment of F0 HCV carriers

We conducted an additional comparison (Table 1) between scenarios *screen-and-treat* versus *screen-and-treat/monitor*. This comparison zooms in on the costs and health gains involved in the immediate treatment of asymptomatic (F0) HCV carriers. Many of these F0 patients would never develop disease if left untreated, and thus in scenario screen-and-treat/monitor they are treated potentially delayed. In all four cohorts, the comparison between scenarios *screen-and-treat/monitor* and *screen-and-treat* yielded very high ICERs, ranging from €56,260 per QALY gained for the *screen-and-treat* scenario for first-time pregnant women and €56,722 per QALY gained for the *screen-and-treat* scenario for all pregnant migrant women. Besides all scenarios for this comparison are not cost-effective, indicating that treatment of F0 HCV carriers is not cost-effective against a WTP-threshold of €20,000 per QALY gained; it is not even cost-effective against a threshold of €50,000 per QALY gained [39].

### Effects of vertical transmission

As indicated above, HCV screening and treatment of pregnant women prevents significant numbers of vertical transmission cases. Yet, the effects of vertical transmission on the ICERs of the *screen-and-treat* and the *screen-and-treat/monitor* scenarios remain limited. This is primarily due to the relatively low rate of vertical transmission of 4.5–7.1% [21]. With inclusion of vertical transmission in the Markov model, the ICERs for the four different cohorts range between €9306 and €10,173, and without inclusion of vertical transmission in the model, the ICERs range between €8780 and €9703.

### Sensitivity analyses

**Fig. 2:**
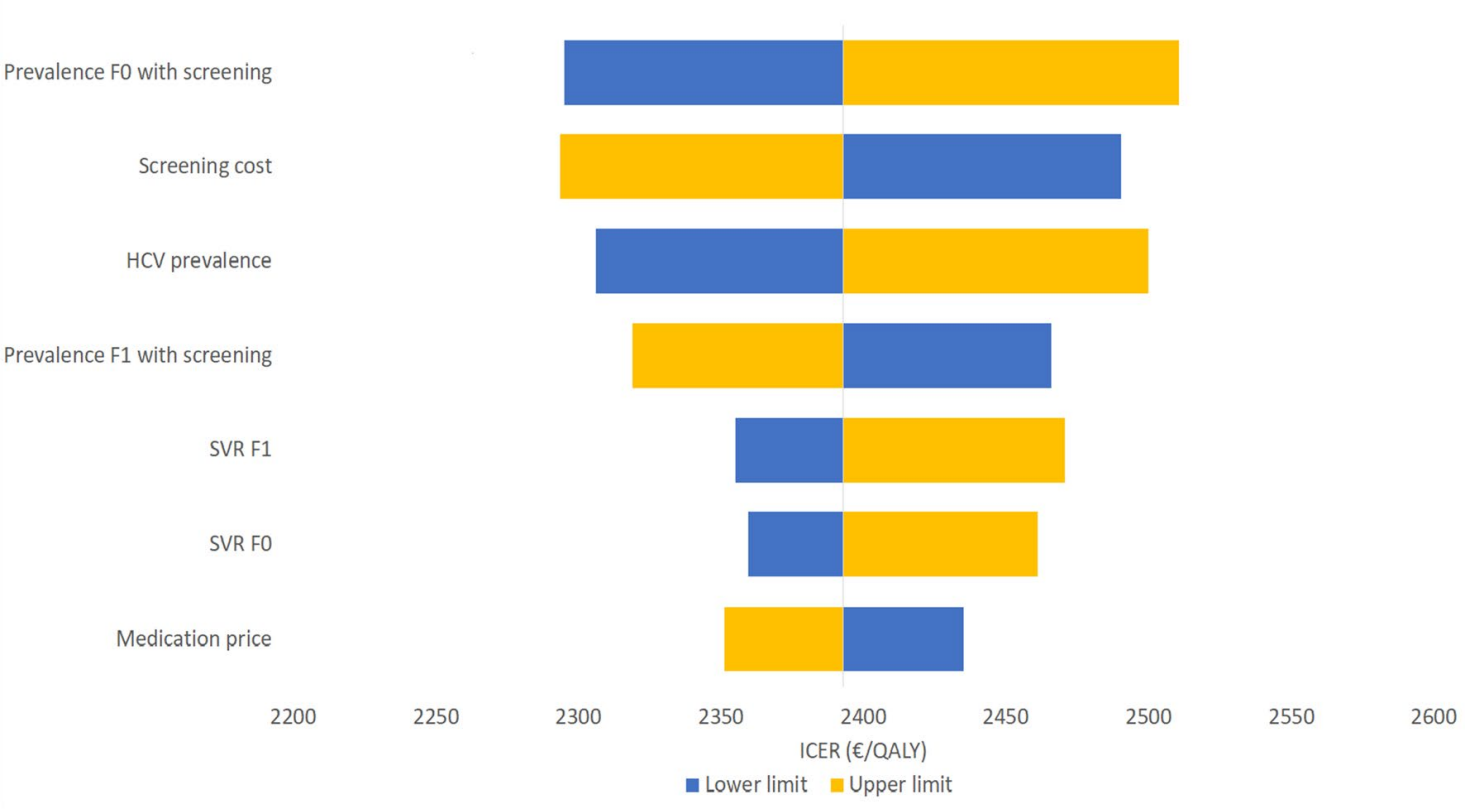
One-way sensitivity analysis for the comparison between the *screen-and-treat/monitor* and *no intervention* scenarios among first-time pregnant migrants. The diagram shows the change in the ICER when each parameter is increased or reduced with 10%

**Fig. 3:**
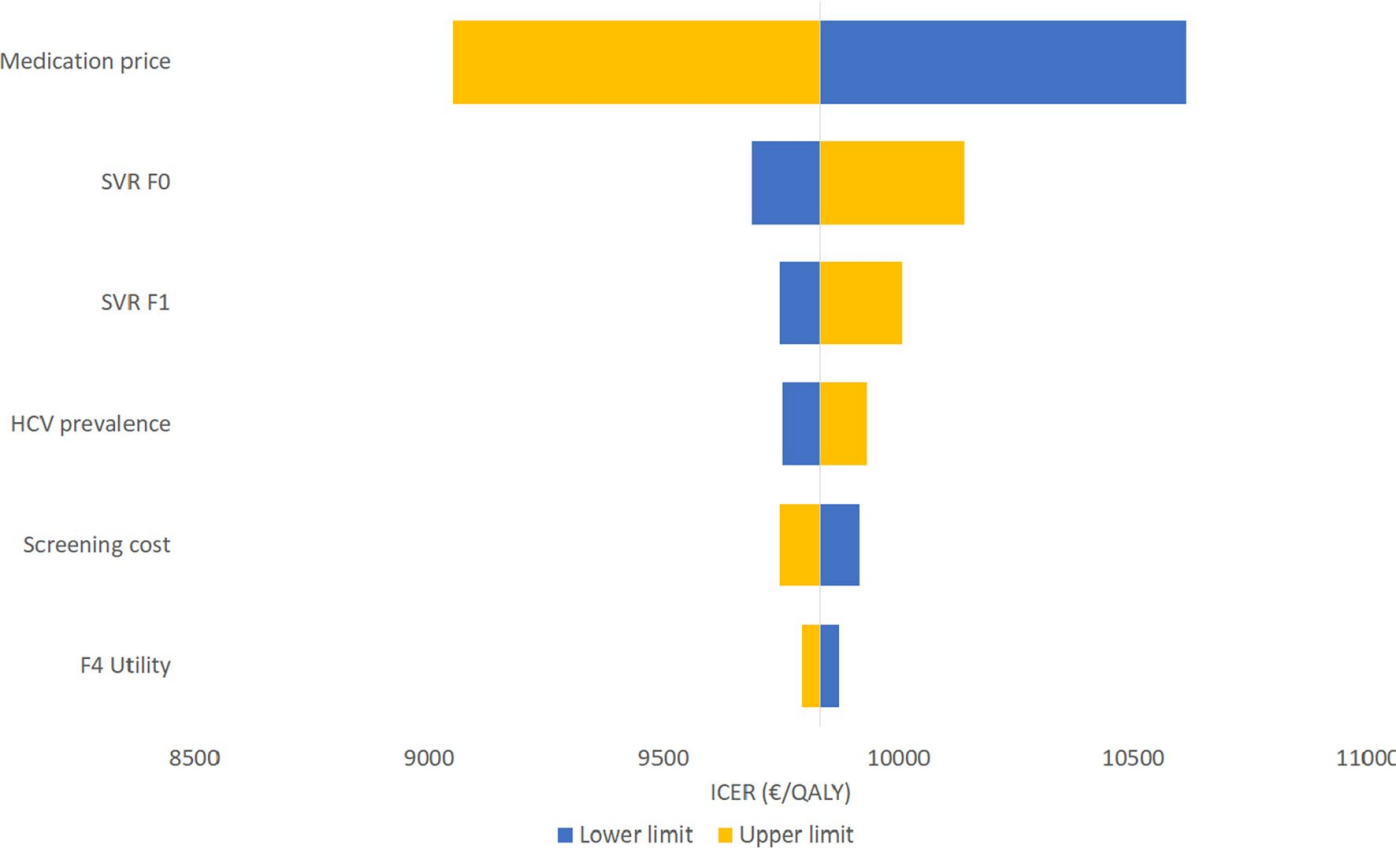
One-way sensitivity analysis for the comparison between the *screen-and-treat* and *no intervention* scenarios among first-time pregnant migrants. The diagram shows the change in the ICER when each parameter is increased or reduced with 10%

**Fig. 4:**
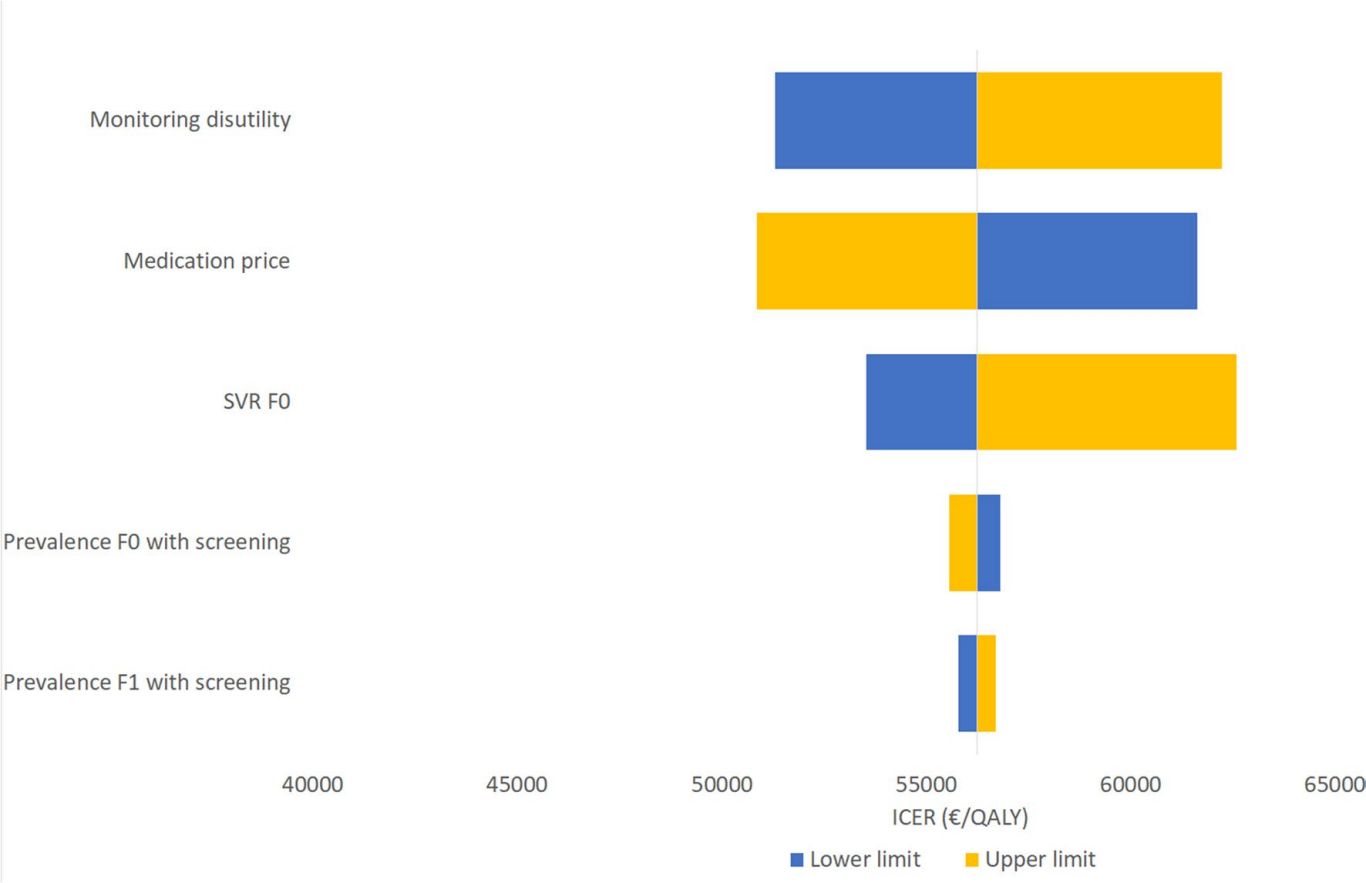
One-way sensitivity analysis for the comparison between the *screen-and-treat* and *screen-and-treat/monitor* scenarios among first-time pregnant migrants. The diagram shows the change in the ICER when each parameter is increased or reduced with 10%

We performed both a one-way sensitivity analysis and a probabilistic sensitivity analysis (PSA), to assess the effect of parameter uncertainty on the cost-effectiveness outcomes. The effect of the cohort size on the ICER outcomes was found to be minimal. Here, we present the result on the univariate sensitivity analysis for the *screen-and-treat/monitor versus no intervention* scenario in the cohort of first-time pregnant migrants. This is the scenario with the most favorable cost-effectiveness. The one-way sensitivity-analysis for this scenario in this cohort shows that the cost-effectiveness outcome is most sensitive to variation in the prevalence of HCV (Fig. 2). For the screen-and-treat versus no intervention scenario in the same cohort, the cost-effectiveness outcome was most sensitive to variation in medication price (Fig. 3). For the screen-and-treat versus screen-and-treat/monitor scenario, monitoring disutility is most sensitive to variation (Fig. 4).

**Fig. 5:**
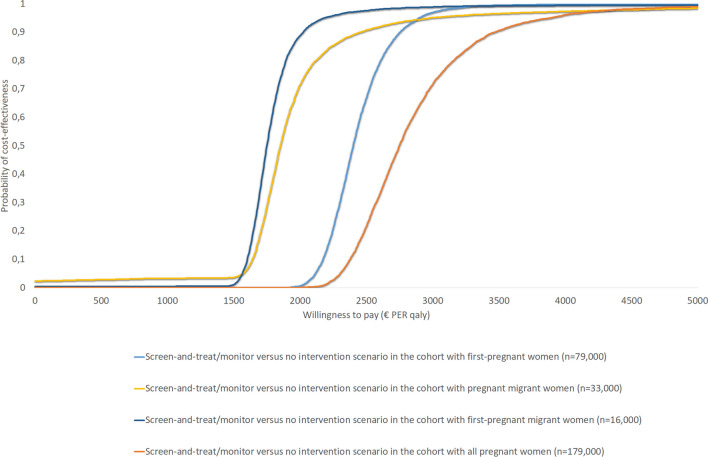
Cost-effectiveness planes for the comparison between the *screen-and-treat/monitor* and *no intervention* scenarios among the four cohorts of pregnant women

**Fig. 6:**
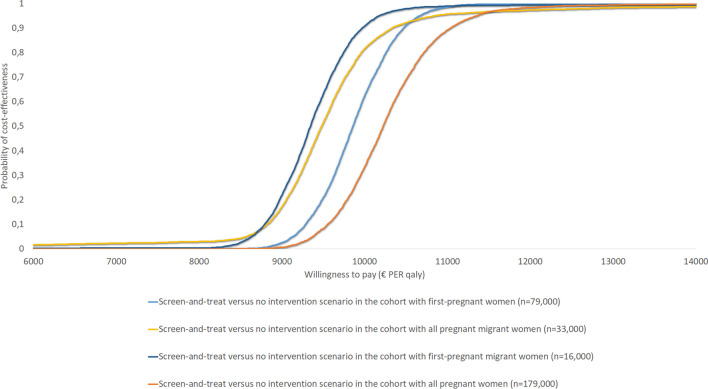
Cost-effectiveness acceptability curve (CEAC) for the comparison between the *screen-and-treat* and *no intervention* scenarios among the four cohorts of pregnant women

The results of the CEAC are presented in Figs. 5 and 6. These results indicate that, among the four cohorts investigated, the ICERs for both the *screen-and-treat* versus *no intervention* and *screen-and-treat/monitor* versus *no intervention* scenarios remain well below the informal Dutch WTP-threshold of €20,000 per QALY gained. Overall, the results of the PSA showed limited variation around the mean cost-effectiveness estimate upon varying the model inputs independently, underlining the robustness of the model.

**Fig. 7:**
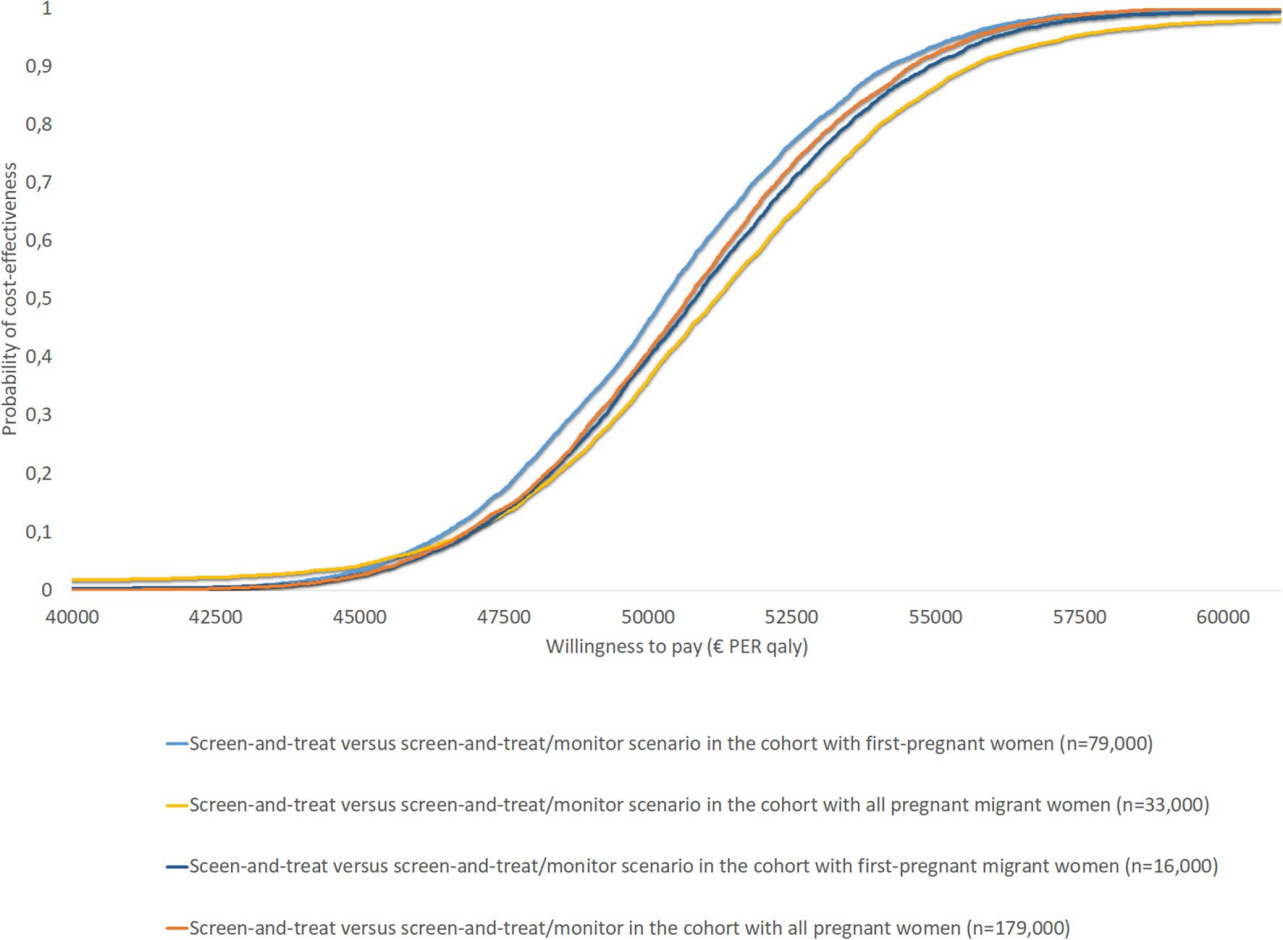
Cost-effectiveness acceptability curve (CEAC) for the comparison between the *screen-and-treat* and *screen-and-treat/monitor* scenarios among the four cohorts of pregnant women

Finally, Fig. 7 shows the respective cost-effectiveness acceptability curve based on varying the WTP-threshold. These results indicate that among the four cohorts investigated, the ICERs for screen-and-treat versus screen-and-treat/monitor is not below the informal Dutch WTP-threshold of €20,000 per QALY gained.

## Discussion

Our study demonstrates that, after screening of pregnant women, identification of HCV patients at early METAVIR stages and implementation of DAA treatment would prevent one out of three liver-related diseases caused by HCV on the long term. In addition, depending on the specific screening/treatment strategy, the size and the HCV prevalence of the cohorts, HCV screening and treatment results in prevention of 10–27 vertical transmissions in the Netherlands.

Our present study demonstrates that HCV screening of pregnant women and subsequent immediate treatment of all HCV-positive individuals with DAAs is a cost-effective intervention in the Netherlands. This applies not only to the cohorts of non-western migrant women in the Netherlands with a relatively high HCV prevalence, but also to the cohorts of all pregnant Dutch women in which on average the HCV prevalence is lower. Indeed, in all four different cohorts studied, the ICERs of the *screen-and-treat* versus *no intervention* scenario were similar, varying between €9306 and €10,173 per QALY gained, and thus remained well below the WTP-threshold of €20,000 per QALY gained.

Still considerably lower ICERs were obtained for the *screen-and-treat/monitor* scenario in which only the symptomatic F1–4 patients are treated and the asymptomatic F0 HCV carriers are just monitored until some of them progress to disease. Indeed, for this scenario the ICERs among the different cohorts varied between only €1739 and €2749 per QALY gained, the most cost-effective result being obtained for the cohort of first-time pregnant migrant women.

While, as indicated above, the ICER of HCV screening and treatment (or monitoring of F0 and treatment of F1–4 patients), remained below the Dutch WTP-threshold of € 20,000, the budget impact of these interventions was substantially different between the four cohorts. Clearly, the budget impact is directly proportional to the size of the cohort, and thus was much higher for the cohorts of all pregnant Dutch women, as opposed to the migrant women. For the *screen-and-treat* scenario, the budget impact varied between €6,283,830 and €19,220,405 in the migrant cohort and all pregnant women, respectively. Also, the extent of treatment strongly affects the budget impact. For example, in the cohort of all pregnant women, the budget impact of the *screen-and-treat/monitor* scenario was, with €5,607,556, much lower than the €19,220,405 of the *screen-and-treat* scenario. Likewise, in the cohort of migrant women, the budget impact varied substantially between these two scenarios, ranging from €1,734,575 and €9,323,994.

The above results illustrate that implementation of a strategy of active monitoring of F0 patients, rather than immediate treatment of these asymptomatic individuals, represents an effective way of reducing the costs of HCV screening and treatment. The reason is that approximately 20% of HCV-infected individuals spontaneously clear the virus, while furthermore 80% of those who do become chronic HCV carriers, will never develop HCV-related liver disease [42]. Clearly, postponing treatment of F0 patients saves potentially unnecessary costs. Accordingly, restriction of treatment to F1–4 patients represents the most cost-effective scenario and thus contributes to optimization of value for HCV patients. This is also illustrated by the comparison of our scenarios *ii* and *iii*, resulting in an ICER above €50,000 per QALY gained in all cohorts studied, which directly demonstrates that treatment of F0 patients is not cost-effective. A 66% DAA discount, in the comparison of *screen-and-treat* versus *screen-and-treat/monitor* all HCV-infected pregnant women, would be cost-effective at a threshold of €20,000 per QALY gained, in different cohorts of pregnant women. 66% discount, is comparable with the discount rate from biologicals versus biosmilars in the Netherlands, therefore in the future screen-and-treat could also be a cost-effective scenario compared to screen and monitoring [43].

While just monitoring of asymptomatic chronic HCV carriers does reduce costs, it does not prevent spread of the virus through vertical transmission from mother-to-child. Monitoring of F0 carriers does not prevent HCV infection in subsequent pregnancies either; our model did not take further transmission of HCV and spreading of infection into account in untreated women. In this respect, our model can be considered to reflect a conservative estimate of cost-effectiveness. Inclusion of transmission effects beyond the child would further enhance the cost-effectiveness profile. However, these effects do not outweigh the benefits of restricting treatment to F1–4 patients. We therefore conclude that monitoring of F0 HCV-positive patients instead of immediate treatment prevents significant costs and thus results in the most favorable cost-effectiveness with a substantially lower budget impact [44].

In this study, we focused on screening of pregnant women and subsequent treatment of HCV-positive individuals with DAAs. However, currently, HCV treatment with DAAs is contraindicated for pregnant women, because of a lack of studies regarding direct teratogenic effects and pharmacological effects later in life of the offspring. Consequently, under the present circumstances, HCV-positive mothers can only be treated after childbirth and thus only children from subsequent pregnancies would be protected. According to Bernstein et al., universal HCV screening and treatment with DAAs during pregnancy is on the horizon [45]. Clearly, these interventions should be urgently evaluated for safety and implemented if appropriate [46]. Several studies regarding DAA treatment of HCV infection during pregnancy are ongoing. For example, the results of a phase I study in Magee Women’s Hospital in Pittsburgh are expected to be presented in 2020 [47]. In the future, we anticipate a development for HCV screening and treatment similar to that in the case of HIV/AIDS, where HIV-positive women are treated with combination antiretroviral therapy (cART) to prevent mother-to-child transmission of the virus [33, 48]. Perinatal transmission is the primary HCV transmission route among children responsible for 70–90% of cases. Many children often remain untested and potentially HCV undiagnosed. Therefore, next to the direct benefit of treatment for the women in curing their infection and preventing serious liver-related diseases, benefits for the child exist in avoiding HCV with possible extrahepatic effects of HCV infection in childhood and significant reductions in both physical and psychosocial health as well as in cognitive functions.

The outcome of our study that HCV screening and treatment of pregnant women in the Netherlands is a cost-effective intervention against the informal Dutch WTP-threshold of €20,000 per QALY gained, is in apparent disagreement with the findings of Urbanus et al. [18] in 2013. These authors estimated that only if costs per treatment were to decline to €3750 (a reduction in price of €31,000), screening of all pregnant women would be cost-effective. However, the results of Urbanus et al*. *[18] were obtained before the introduction of the highly effective DAAs in 2015. Now, it appears that screening and DAA treatment, of HCV-positive individuals would be a cost-effective intervention. Nonetheless, as discussed above, screening of the entire population of pregnant women is not necessarily preferred, because of the large budget impact of the intervention and the low HCV prevalence in the total Dutch population. Kracht et al*.* have proposed “micro-elimination” of HCV by screening and treatment of various pre-defined HCV risk groups [49]. These authors concluded, in agreement with our results, that HCV screening of risk groups is the most pragmatic and efficient approach.

Our study could be helpful with decisions on the implementation of HCV screening programmes in Europe. The estimated fraction of HCV cases that remain undiagnosed in the general or proxy populations in Europe ranges between 20% in Denmark to 91.2% in Greece [50]. Razavi et al. estimated the overall proportion of undiagnosed HCV cases in the EU at 64% [1]. DAA treatment of HCV in pregnancy is not (yet) in clinical guidelines, our model is hypothetical currently in that respect. The main difference between, for example, the ASSLD/IDSA-guidelines and our model is that we assumed that pregnant women are treated with DAAs after HCV diagnosis during pregnancy. A simplified treatment algorithm [51], for treatment-naive patients without cirrhosis, possibly would reduce the costs in the model, which could also improve the performance of treatment, corresponding with favorable to cost-effectiveness [52].

This study reflects a single cohort model in the Netherlands, with effects on children for that specific cohort. Our current analysis does not include future pregnancies in the very same cohorts. In the future, the total amounts of screened and treated pregnant women will be lower and preferably result from the standard prenatal screening for infectious diseases, which means higher numbers to screen to identify patients, but also less patients to be treated with relatively expensive treatments. Our study demonstrates that screening and monitoring or treatment of smaller subgroups of pregnant women is highly cost-effective approach and has a comparatively low budget impact in The Netherlands. On the other hand, in other countries with a higher HCV prevalence, screening of all pregnant women could be a more cost-effective option [6, 53].

## Conclusions

Our study indicates that universal HCV screening of pregnant women in the Netherlands is cost-effective, independent of the specific cohort involved. However, the budget impact is substantially different between subgroups, and is largely determined by the cohort size and by the extent of treatment of HCV-positive individuals. Screening and subsequent monitoring of F0 patients and treatment of F1-F4 patients with the DAAs appeared to be the most cost-effective approach. HCV screening and treatment of pregnant women results in a substantial reduction of HCV-related liver diseases and deaths. It also prevents vertical transmission of the virus from mother to child. From a public-health and health-economic perspective, it would be reasonable to consider smaller risk groups of first-time pregnant or/and non-western pregnant women for an active HCV screening programme in the Netherlands, and possibly elsewhere.

## Electronic supplementary material

Below is the link to the electronic supplementary material.

Supplementary file1 (DOCX 33 kb)

Supplementary file2 (DOCX 28 kb)
