# Supplementary material for: Cost-effectiveness of hepatitis C virus screening, and subsequent monitoring or treatment among pregnant women in the Netherlands
Source: Eur J Health Econ. 2020 Oct 16;22(1):75–88. doi: 10.1007/s10198-020-01236-2 (PMC7561704; doi:10.1007/s10198-020-01236-2)
Supplement: Supplementary file 2 — Supplementary file2 (DOCX 28 kb) [file 10198_2020_1236_MOESM2_ESM.docx]

| S2 Appendix: PSA parameters variation | | | | |
| --- | --- | --- | --- | --- |
| *Parameter* | | ***Value*** | ***Low*** | ***High*** |
| Prevalence | |  |  |  |
| Pregnant women | | 0.0026 (n=465) [4] | 0.0015 | 0.0046 |
| First-time pregnant women | | 0.0037 (n=292) ^assumed^ | 0.0029 | 0.0044 |
| Pregnant migrants | | 0.0070 (n=231) [1-3] | 0.0056 | 0.0084 |
| First-time pregnant migrants | | 0.0100 (n=160) ^assumed^ | 0.0080 | 0.0120 |
| Distribution of Metavir stage with and without screening | |  |  |  |
| Metavir score screening F0 | | 0.89 ^assumed^ | 0.801 | 0.979 |
| Metavir score screening F1 | | 0.08 ^assumed^ | 0.072 | 0.088 |
| Metavir score screening F2 | | 0.02 ^assumed^ | 0.018 | 0.022 |
| Metavir score screening F3 | | 0.008 ^assumed^ | 0.0072 | 0.0088 |
| Metavir score screening F4 | | 0.002  ^assumed^ | 0.0018 | 0.0022 |
| Metavir score No Screening F0 | | 0.17 [5] | 0.14 | 0.19 |
| Metavir score No Screening F1 | | 0.35 [5] | 0.26 | 0.39 |
| Metavir score No Screening F2 | | 0.22 [5] | 0.18 | 0.24 |
| Metavir score No Screening F3 | | 0.14 [5] | 0.12 | 0.15 |
| Metavir score No Screening F4 | | 0.12 [5] | 0.11 | 0.13 |
| Costs | |  |  |  |
| Serological test | | €12.69 [6] | 11.421 | 13.959 |
| PCR test | | €122 [7] | 109.8 | 134.2 |
| Fibroscan | | €236 [7] | 212.4 | 259.6 |
| Monitoring | | €100 [8] | 90 | 110 |
| SVR F0-F3 | | € 426 [9] | 383.4 | 468.6 |
| SVR F4 | | € 673 [9] | 605.7 | 740.3 |
| F4 | | € 821 [9] | 738.9 | 903.1 |
| F4 | | € 821 [9] | 738.9 | 903.1 |
| DCC | | € 27,921 [9] | 25128.9 | 30713.1 |
| HCC | | € 21,054 [9] | 18948.6 | 23159.4 |
| LT | | € 143,226 [9] | 128903.4 | 157548.6 |
| Post LT | | € 20,714 [9] | 18642.6 | 22785.4 |
| Post LT 2 | | € 20,714 [9] | 18642.6 | 22785.4 |
| Baseline utilities | |  |  |  |
| F0 | | 0.97 [10,11] | 0.873 | 1 |
| F1-F2 | | 0.95 [10,11] | 0.7 | 1 |
| F3 | | 0.85 [10,11] | 0.66 | 1 |
| F4 | | 0.79 [10,11] | 0.46 | 1 |
| DC | | 0.72 [10,11] | 0.26 | 0.91 |
| HCC | | 0.72 [10,11] | 0.15 | 0.95 |
| LT | | 0.5 [10,11] | 0.2 | 0.8 |
| PostLT | | 0.7 [10,11] | 0.2 | 0.8 |
| Post LT + | | 0.825 [10,11] | 0.64 | 1 |
| Distribution of HCV by age | |  |  |  |
| <30 | | 0.6 ^assumed^ | 0.54 | 0.66 |
| 30-39 | | 0.38 ^assumed^ | 0.342 | 0.418 |
| 40-49 | | 0.02 ^assumed^ | 0.018 | 0.022 |
| Transition state | |  |  |  |
| From | to | Transition probabilities |  |  |
| Treatment | SVR F0 | 0.95 [12] | 0.9 | 0.98 |
|  | SVR F1 | 0.95 [12] | 0.9 | 0.98 |
|  | SVR F2 | 0.95 [12] | 0.9 | 0.98 |
|  | SVR F3 | 0.95 [12] | 0.9 | 0.98 |
|  | SVR F4 | 0.9 [12] | 0.85 | 0.95 |
| F0-F3 | F4 | 0.073 [13] | 0.01 | 0.232 |
| F4 | DCC | 0.039 [13] | 0.02 | 0.083 |
|  | HCC | 0.037 [13] | 0.01 | 0.044 |
|  | Death | 0.053 [13] | 0.04 | 0.06 |
| DCC | HCC | 0.037 [13] | 0.01 | 0.044 |
|  | LT | 0.03 [13] | 0.01 | 0.062 |
|  | Death | 0.13 [14] | 0.065 | 0.193 |
| HCC | Death | 0.43 [15,16] | 0.33 | 0.86 |
| LT | Post LT1 | 0.79 [15,16] | 1 | 1 |
|  | Death | 0.21 [15,16] | 0.06 | 0.42 |
| Post LT1 | Post LT+ | 0.943 [15,16] | 1 | 1 |
|  | Death | 0.057 [15,16] | 0.024 | 0.11 |
| POST LT+ | Death | 0.02 [15,16] | 0.012 | 0.042 |
| F0 | F1 | 0.117 [16] | 0.107 | 0.127 |
| F1 | F2 | 0.085 [16] | 0.078 | 0.093 |
| F2 | F3 | 0.121 [16] | 0.112 | 0.13 |
| F3 | F4 | 0.115 [16] | 0.107 | 0.123 |

***METAVIR score: F0.F1.F2.F3.F4. SVR: Sustained Virologic response. HCC: hepatocellular cancer. DC: Decompensated cirhossis.LT: liver transplantation. LRD: Liver related death***

**References**

1. Page Axley, Zunirah Ahmed, Sujan Ravi, and Ashwani K. Singal. Hepatitis C Virus and Hepatocellular Carcinoma: A Narrative Review. J Clin Transl Hepatol. 2018 Mar 28; 6(1): 79–84.
2. European Association for the Study of the Liver: EASL: recommendations on Treatment of Hepatitis C 2016. Geneva, Switzerland, summary september 2016.
3. Gezondheidsraad, Health council of the Netherlands. Screening van risicogroepen op hepatitis B en C. Den Haag: Gezondheidsraad, executive summary no. 2016/6: 27-28.
4. Urbanus AT, van de Laar TJ, van den Hoek A, Zuure FR, Speksnijder AG, Baaten CG e.a. Hepatitis C in the general population of various ethnic origins living in the Netherlands: should non- western migrants be screened? J Hepatol 2011; 55(6): 1207-1214
5. Urbanus AT, van Keep M, Matser AA, Rozenbaum MH, Weegink CJ, van den Hoek A, et al. Is Adding HCV Screening to the Antenatal National Screening Program in Amsterdam, The Netherlands, Cost-Effective? Jhaveri R, editor. PLoS One. 2013 Aug 12;8(8):e70319.
6. Liu S, Schwarzinger M, Carrat F, Goldhaber-Fiebert JD. Cost Effectiveness of Fibrosis Assessment Prior to Treatment for Chronic Hepatitis C Patients. Jhaveri R, editor. PLoS One. 2011 Dec 2;6(12):e26783.
7. Afdhal NH. Fibroscan (transient elastography) for the measurement of liver fibrosis. Gastroenterol Hepatol (N Y). 2012 Sep;8(9):605–7.
8. Richtlijn voor het uitvoeren van economische evaluaties in de gezondheidszorg | Publicatie | Zorginstituut Nederland [Internet]. [cited 2017 Sep 20]. Available from: https://www.zorginstituutnederland.nl/over-ons/publicaties/publicatie/2016/02/29/richtlijn-voor-het-uitvoeren-van-economische-evaluaties-in-de-gezondheidszorg
9. Liu S, Cipriano LE, Holodniy M, Owens DK, Goldhaber-Fiebert JD. New Protease Inhibitors for the Treatment of Chronic Hepatitis C. Ann Intern Med. 2012 Feb 21;156(4):279.
10. McLernon DJ, Dillon J, Donnan PT. Systematic Review: Health-State Utilities in Liver Disease: A Systematic Review. Med Decis Mak. 2008 Jul 18;28(4):582–92.
11. WHO | Guidelines for the screening, care and treatment of persons with chronic hepatitis C infection. WHO. 2016;
12. Younossi ZM, Singer ME, McHutchison JG, Shermock KM. Cost effectiveness of interferon ?2b combined with ribavirin for the treatment of chronic hepatitis C. Hepatology. 1999 Nov;30(5):1318–24.
13. Salomon JA. Cost-effectiveness of Treatment for Chronic Hepatitis C Infection in an Evolving Patient Population. JAMA. 2003 Jul 9;290(2):228.
14. Interferon alpha (pegylated and non-pegylated) and ribavirin for the treatment of mild chronic hepatitis C: a systematic review and economic evaluation - PubMed - NCBI.
15. Plunkett BA, Grobman WA. Routine hepatitis C virus screening in pregnancy: A cost-effectiveness analysis. Am J Obstet Gynecol. 2005 Apr;192(4):1153–61.
16. Thein H-H, Yi Q, Dore GJ, Krahn MD. Estimation of stage-specific fibrosis progression rates in chronic hepatitis C virus infection: A meta-analysis and meta-regression. Hepatology. 2008 Aug 1;48(2):418–31.
